# Th1/Th2 Balance and Th17/Treg-Mediated Immunity in relation to Murine Resistance to Dextran Sulfate-Induced Colitis

**DOI:** 10.1155/2017/7047201

**Published:** 2017-05-11

**Authors:** Fangli Yang, Danan Wang, Yan Li, Lixuan Sang, Junfeng Zhu, Jinyan Wang, Bing Wei, Changlong Lu, Xun Sun

**Affiliations:** ^1^Department of Immunology, China Medical University, Shenyang 110122, China; ^2^Department of Immunology, College of Basic Medical Sciences, Shenyang Medical College, Shenyang 110034, China

## Abstract

**Background:**

The role of the Th17/Treg balance in the development of experimental colitis remains poorly understood.

**Methods:**

We exploited the differential response of BALB/c mice and C57BL/6 mice towards drinking water mediated by dextran sulfate sodium (DSS) challenge.

**Results:**

DSS-resistant BALB/c mice were characterized by low levels of IFN-*γ* and TNF-*α* but high levels of IL-4, IL-6, IL-10, IL-17A, IL-17F, and colon lamina propria and mesenteric lymph node (MLN) CD4^+^CD25^+^FoxP3^+^ T cells when compared to C57BL/6 mice. Collectively, these data indicate the propensity of BALB/c mice towards a Th2/Th17/Treg-polarized immunity protecting these animals against DSS challenge, whereas Th1-polarization of C57BL/6 mice confers sensitivity to DSS-induced colitis.

**Conclusions:**

The intrinsic congenital capacity of mouse strains with respect to T cell proliferation determines sensitivity to experimental colitis.

## 1. Introduction

The inflammatory bowel diseases (IBD), which include ulcerative colitis (UC) and Crohn's disease (CD), are characterized by chronic intestinal inflammation provoked by an aberrant innate and/or adaptive immunity against the bacterial flora in a genetically predisposed host [1, 2], but whose etiology remains largely obscure [3]. In view of the increasing incidence of IBD, studies to elucidate the mechanisms driving differential susceptibility towards IBD development are urgently needed. Indeed, T cells and their secreted cytokines are the main effectors in the induction and perpetuation of intestinal inflammation [4]. The available evidence suggests that CD is characterized with a Th1 response, while UC is associated with an atypical Th2 response [5].

In addition to the classical Th1 and Th2 responses, a role for IL-17-expressing CD4^+^T lymphocyte (Th17 cells) response has also emerged [6]. Th17 cells are present in all segments of the intestine including the large and small intestine [7]. They are naturally resident in the intestinal lamina propria, where they develop in a specific cytokine and microbial milieu [8, 9]. Th17 cell cytokines are a characteristic of inflammatory bowel disease [10–13]. The regulatory T cells (Tregs) are an additional T cell subset potentially involved in the reaction of the intestine to colitis-provoking stimuli [14]. Based on studies in which mucosal Tregs are depleted, Tregs were found to play a key role in the maintenance of gut mucosal homeostasis by suppressing abnormal immune response against the commensal flora or dietary antigens. Whether intrinsic interindividual differences in the capacity to support a Th17 and Treg responses explain differential susceptibility towards inflammatory bowel disease remains, however, unexplored.

The nature of T cell response appears fundamental in steering the natural course of IBD. However, direct experimental demonstration that intrinsic properties of the T cell compartment mediates the sensitivity to suffer from colitis has not been provided.

Genetic background is a key factor in determining the susceptibility of experimental rodents to DSS colitis, as evident from the existence of marked species and strain differences [15, 16]. The reasons underlying strain differences remain unclear but offer an opportunity to address the potential influence of intrinsic properties of T cell compartment on the susceptibility to develop mucosal inflammation. Such studies may help translating preclinical findings into clinical practice and thus of utmost importance for future designing and understanding the results from immunological research. In the present study, we compared the reaction of different T cell compartments and associated cytokine production against DSS challenge in DSS-resistant BALB/c mice and DSS-induced colitis permissive C57BL/6 mice. The results indicate that Th17/Treg responses are important in preventing the development of colitis.

## 2. Materials and Methods

### 2.1. Materials

DSS (MW 36,000–50,000) was purchased from MP Biomedicals (USA). Antibodies for flow cytometry analysis were purchased from BD Biosciences (San Diego, CA, USA) or BioLegend (San Diego, CA, USA). Propidium iodide, phorbol-12-myristate-13-acetate (PMA), and ionomycin were obtained from Sigma-Aldrich (USA).

### 2.2. Induction and Evaluation of Acute Colitis by DSS

Reporting of this work adhered to ARRIVE guidelines; the corresponding checklist is available as Supplementary Data available online at https://doi.org/10.1155/2017/7047201 [17]. Male BALB/c mice and C57/BL6 mice were purchased from Liao Ning Chang Sheng Biotechnology Co., Ltd. (Liaoning, China). All mice were maintained under specific pathogen-free conditions and offered food and water ad libitum. All studies were performed in accordance with the China Medical University Animal Care and Use Committee guidelines under routine husbandry conditions. Animals belonging to the same experimental group were housed in a single cage, and animals were maintained in their home cage throughout the experiment up to sacrifice. As the study concerns strain comparison, randomisation was not applicable. For acute colitis induction by DSS, mice were administered 3.5% (wt/vol) of DSS in drinking water in the morning, which was provided ad libitum for 6 days. Body weight and the DAI [18] were evaluated daily. Mice were sacrificed on day 7, and colon length was measured. The middle parts from colon were fixed in 4% paraformaldehyde and performed with H&E. Histology was scored as described previously [19]. Apart from Kaplan-Meier analysis, dead animals were ignored in the other analyses.

### 2.3. Enzyme-Linked Immunosorbent Assay (ELISA)

Lamina propria lymphocytes (LPLs) in the colon were isolated using a previously described method [20]. Isolated LPLs were stimulated by incubation for 48 h in vitro in 96-well flat-bottom plates coated with soluble anti-CD3 (10 *μ*g/ml) and anti-CD28 (2 *μ*g/ml) antibodies in 0.2 ml RPMI 1640 containing 10% FBS. TNF-*α*, IFN-*γ*, IL-4, IL-5, IL-6, IL-10, IL-17A, IL-17F, and IL-22 concentrations were determined using ELISA kits (R&D Systems).

### 2.4. Flow Cytometry

Specific cell populations were identified by incubating the LPLs and mononuclear cells from MLN with an Fc*γ*R-blocking mAb and indicated fluorescently labeled antibodies at 4°C for 30 min. For intracellular cytokine staining, freshly isolated LPLs were analyzed by flow cytometry using a Cytofix/Cytoperm Kit Plus (BD Biosciences, San Jose, CA). LPLs were stimulated with PMA (25 ng/ml; Sigma-Aldrich, St. Louis, MO) and ionomycin (1 *μ*g/ml; Sigma-Aldrich) for 5 h at 37°C. Brefeldin A (10 *μ*g/ml; Sigma-Aldrich) was added for the last 4 h of incubation. These cells were then stained with mAbs against mouse IL-17A or IFN-*γ* for 30 min at 4°C. To determine Treg cells, LPLs and MLNs were analyzed by flow cytometry using a Mouse Regulatory T Cell Staining Kit (BD Biosciences, San Jose, CA).

### 2.5. Real-Time Quantitative Polymerase Chain Reaction (RT-PCR)

Total RNA was extracted from colon and MLN of mice using the RNAiso plus (Takara, Dalian, China). We used 1 *μ*g of total RNA of each mouse for performing reverse transcription with a PrimeScript™ RT reagent kit and gDNA Eraser (Perfect Real Time, Takara) to generate the first strand cDNA. The PCR mixture was prepared using SYBR^®^ Premix Ex Taq™ (Tli RNaseH Plus, Takara) and one of the following primers (see Table 1 for primer sequences).

### 2.6. Statistical Analysis

The difference in survival rates was evaluated by the log-rank test (Mantel–Cox). Differences in parametric data were evaluated by one-way analysis of variance (ANOVA) test. Differences of *p* < 0.05 were considered statistically significant. In all assays, the physical (day 0) cell number of immune cell subtypes is 200,000/sample.

## 3. Results

### 3.1. Differential Susceptibility of C57BL/6 and BALB/c Mice to DSS-Induced Acute Colitis

**Figure 1:**
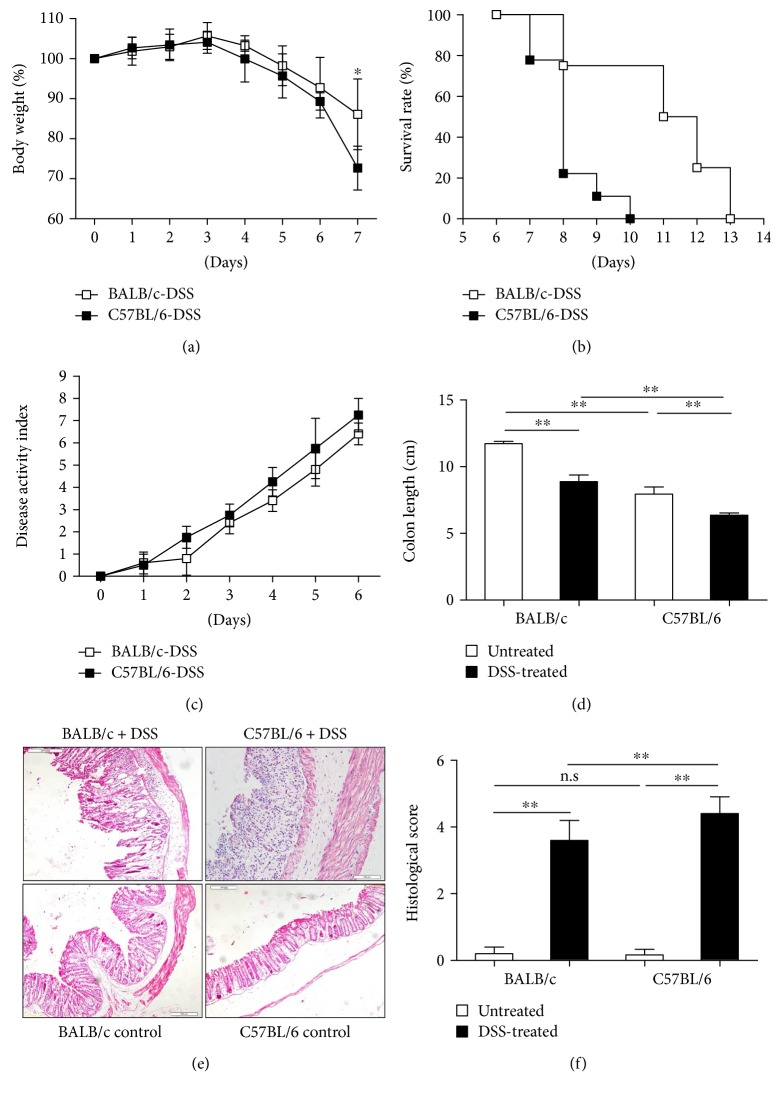
Compared to C57BL/6 mice, BALB/c mice are resistant to DSS-induced mucosal damage and development of acute colitis. Weight loss, survival rate, disease activity index (DAI), colon length, macroscopic changes, and H&E staining of the colon (original magnification ×200) are shown. (a) Weight changes following DSS challenge in C57BL/6 and BALB/c mice. (b) Kaplan-Meier survival curves of BALB/c and C57BL/6 mice following exposure to 3.5% DSS in drinking water (*n* = 10). (c) DAI and (d) colon length changes. (e) Macroscopic changes. (f) H&E staining of colon (original magnification ×200). ^∗^*p* < 0.05; ^∗∗^*p* < 0.01.

Previous studies [15, 21] suggest marked differences in the sensitivity to DSS-induced acute colitis between different mouse strains, suggesting that comparing the immune system of alternative strains can provide clues as determinants that govern alternative susceptibility to intestinal inflammation in general. A first indication that such an approach is indeed feasible came from experiments in which we compared the DSS-induced acute colitis between BALB/c mice and C57BL/6 mice. To this end, healthy C57BL/6 and BALB/c mice were given 3.5% DSS in their drinking water ad libitum for 6 days. As shown in Figure 1, both mouse strains reacted to the DSS challenge with colonic mucosal injury and a concomitant intestinal inflammatory response, which was especially marked in the colon. The induction of colonic epithelial damage in C57BL/6 mice was associated by substantial diarrhea, loss of fecal consistency, and the appearance of visible stool blood as well as weight loss and diminished survival. Strikingly, despite the induction of generalized epithelial damage, these effects were much less prominent in BALB/c mice (Figures 1(a) and 1(b)). In apparent agreement, although DAI was significantly elevated in both mouse strains following DSS challenge, the DAI in C57BL/6 mice was substantially higher than that of BALB/c mice (Figure 1(c)). In addition, shortening of the colon length is considered a good proxy measure for the severity of colitis and DSS-treated C57BL/6 mice show substantial more reduction in this parameter than BALB/c mice (Figure 1(d)). Finally, H&E examination of mouse intestine after DSS induction shows significant pathological changes in the colon of both mouse strains, including prominent mucosal damage and epithelial necrosis. However, only in C57BL/6 mice these epithelial changes are associated by substantial inflammatory damage as judged by appearance of focal crypt lesions, goblet cell loss, and inflammatory cell infiltration (Figures 1(e) and 1(f)). These data show that DSS-induced mucosal damage is associated by a substantial inflammatory response in C57BL/6 mice, but that the immune system of BALB/c mice is much less prone to a fulminant reaction to the DSS challenge.

### 3.2. Differential Cytokine Production by Colonic CD4^+^ T-LPL Cells following DSS Challenge

**Figure 2:**
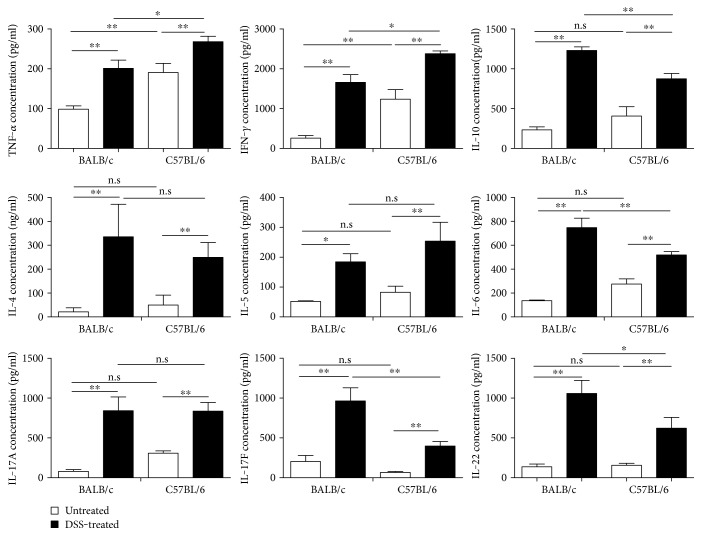
Cytokine production by T-LPLs in DSS-induced acute colitis model of BALB/c and C57BL/6 mice. Cytokine production in the supernatant of anti-CD3*ε*-stimulated cultured LPLs was analyzed by ELISA. Data indicate mean ± SD obtained from two independent experiments, in triplicate each. The levels of IL-1b and IL-23 in colon tissues were below detection limit and thus not shown. Statistical differences were evaluated by Student's *t*-test. ^∗^*p* < 0.05; ^∗∗^*p* < 0.01.

To investigate the mechanistic basis of differential induction of mucosal inflammation in C57BL/6 mice versus BALB/c mice, the levels of TNF-*α*, IFN-*γ*, IL-4, IL-5, IL-6, IL-10, IL-17A, IL-17F, and IL-22 production in the supernatant of LPL cultured with immobilized anti-CD3*ε* mAb were analyzed by ELISA (Figure 2). Compared to BALB/c mice, C57BL/6 mice had significantly higher levels of TNF-*α* and IFN-*γ* in the supernatant of cultured LPL (*p* < 0.05), suggesting a more Th1 dominant immune profile in mice following DSS challenge. Conversely, the DSS-resistant BALB/c mice displayed significantly higher IL-6, IL-17F, and IL-22 in the supernatant of cultured LPL (*p* < 0.05), suggesting that BALB/c mice react to DSS-triggered mucosal damage with a Th2/Th17-dominant immune response (Figure 2). Levels of other cytokines, including IL-4, IL-5, IL-10, and IL-17A, differ significantly between the two models (Figure 2). Thus, our data suggests that DSS-induced mucosal damage is associated with a propensity to develop Th1 response, whereas Th2/Th17-mediated immunity appears to confer protection in this respect.

### 3.3. Differential Immune Cell Response in the Colonic Lamina Propria of BALB/c and C57BL/6 Mice following DSS Challenge

**Figure 3:**
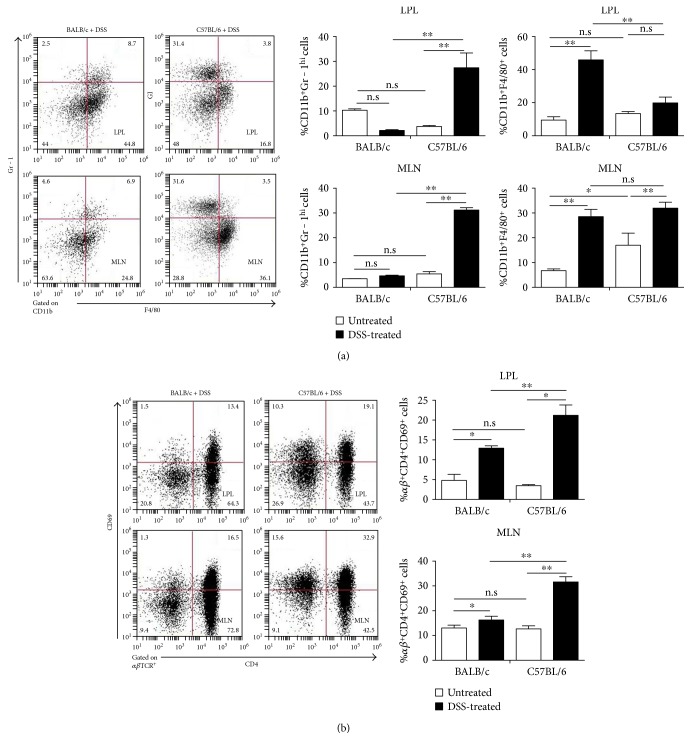
Lymphocyte subsets of LPL and MLN in DSS-challenged BALB/c and C57BL/6 mice as measured by FACS. Data indicate the mean ± SD of three animals, representative of two independent experiments. Statistical significant differences were evaluated by Student's *t*-test. ^∗^*p* < 0.05; ^∗∗^*p* < 0.01. The mononuclear cells were harvested from LP and MLN 6 days after DSS induction and strained with various combinations of mAbs (neutrophil: CD11b^+^Gr-1^high^; monocyte: CD11b^+^F4/80^+^; activated T cells: CD4^+^TCR*αβ*^+^CD69^+^). In all assays, the physical (day 0) cell number of immune cell subtypes is 200,000/sample. (a), (b) The isotype stainings were performed using a different flow cytometer and thus cannot be directly compared and hence are not shown.

The role of differential immune activation as a mechanistic basis for the dichotomy in the reaction to DSS between C57BL/6 and BALB/c mice is supported by experiments characterizing the size of various lymphocyte subcompartments. As evident from Figure 3, the response of C57BL/6 to DSS involves markedly stronger lymphocyte recruitment in general. The percentage of neutrophil and CD4^+^CD69^+^ lymphocytes were significantly lower, but the percentage of macrophage was significantly higher in the lamina propria and MLN of BALB/c colitis mice than C57BL/6 colitis mice. These results are broadly consistent with differential Th1 and Th2 response to DSS between the two mouse strains.

### 3.4. Differential Cytokine mRNA Expression in Acute DSS Colitis in C57BL/6 and BALB/c Mice

**Figure 4:**
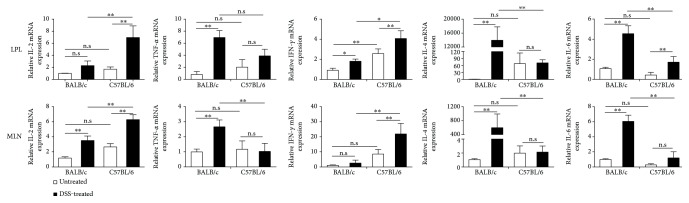
Real-time PCR determination of the cytokine mRNA expression of LPL and MLN following DSS challenge in BALB/c and C57BL/6 mice. Data indicate the mean ± SD obtained from a representative of two independent experiments each consisting of four animals. Statistical significant differences were evaluated by Student's *t*-test. ^∗^*p* < 0.05; ^∗∗^*p* < 0.01. In all assays, the physical (day 0) cell number of immune cell subtypes is 200,000/sample.

The notion that intrinsic differences between the mouse strains to mount a specific immune response underlie the difference in the reaction of C57BL/6 and BALB/c mice towards the DSS challenge was further supported by measuring the mRNA levels; as shown in Figure 4, the DSS-resistant BALB/c mucosal immunity was accompanied by a relatively strong expression of TNF-*α*, IL-4, and IL-6 while the fulminant inflammatory response to DSS in C57BL/6 was associated with strong IL-2 and IFN-*γ* mRNA levels, which again show a Th1-dominated immune reaction in the C57BL/6 mice, but more Th2-polarized immunity in the BALB/c mice. Thus, the differences in cytokine levels observed derive from intrinsic properties with respect to gene expression in the local immune cells.

### 3.5. Differential Th17 and Treg Response in BALB/c and C57BL/6 Mice following DSS Challenge

**Figure 5:**
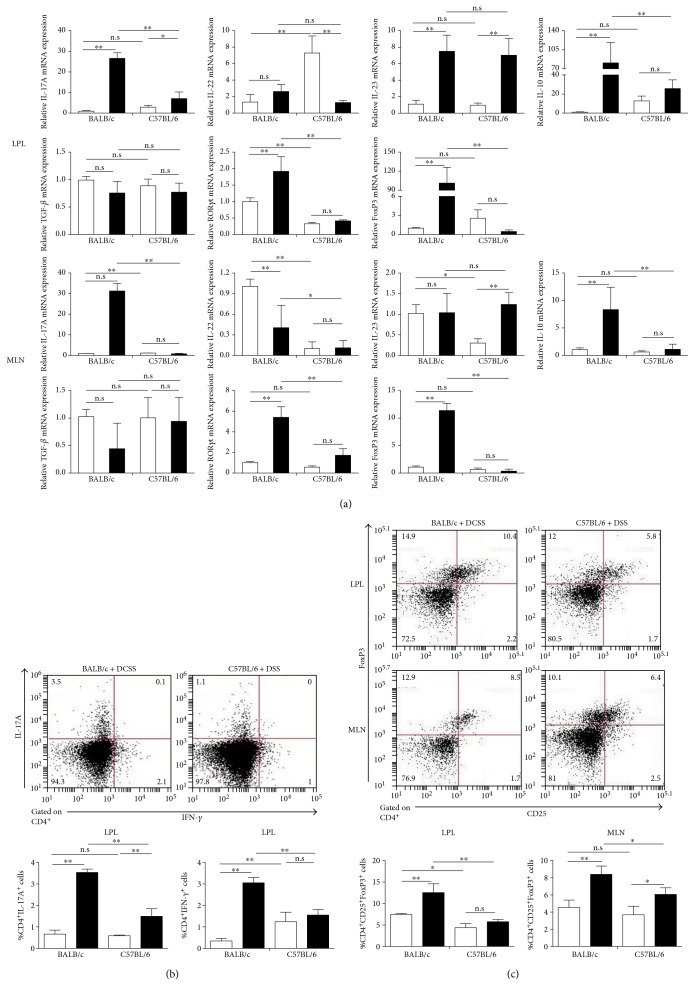
A differential induction of Th17 and Treg responses in BALB/c and C57BL/6 mice following DSS challenge. (a) mRNA expression of various cytokines in LPLs and MLNs was analyzed by RT-PCR. Levels of mRNA were normalized to *β*-actin mRNA and expressed relative to unchallenged BALB/c mice. Data indicate the mean ± SD obtained from a representative of two independent experiments (each with *n* = 4). (b), (c) LPLs (MLN) were stained for cytokines and key nuclear immune factors and analyzed by FACS. Representative plots (left panel) and associated quantification displaying the mean ± SD values of the percentage of Th17 and Treg cells (right panel) are shown (*n* = 3 mice per group). The data are representative of two independent experiments. Statistical differences were evaluated by Student's *t*-test. ^∗^*p* < 0.05; ^∗∗^*p* < 0.01. In all assays, the physical (day 0) cell number of immune cell subtypes is 200,000/sample.

We further explored the nature of the Th17 immunity observed in our experimental mice, by assessing the cell accumulation and mRNA expression of a large panel of Th17-related cytokines in colonic tissue and MLN. As shown in Figure 5, the DSS-resistant BALB/c mucosal immunity was accompanied by a relatively strong expression of IL-17A mRNA, as well as higher percentage of CD4^+^IL-17A^+^ T cells and CD4^+^IFN-*γ*^+^ T cells. In addition, higher FoxP3 and IL-10 mRNA expression and higher percentage of CD4^+^CD25^+^FoxP3^+^ regulatory T cells were detected within the lamina propria and MLN of DSS-resistant BALB/c compared to DSS-sensitive C57BL/6 mice. The activation of Th17 response coincides with a concomitant induction of regulatory T cells, and production of the associated cytokines supports the notion that activation of Th2/Th17 cytokines and also Treg induction is a characteristic of tolerance to a DSS challenge.

### 3.6. Differential Induction of Chemokine mRNA Expression following DSS Challenge of C57BL/6 and BALB/c Mice

**Figure 6:**
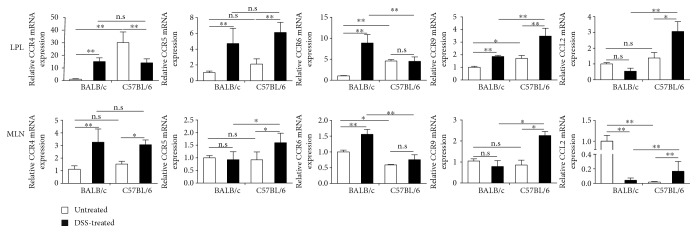
RT-PCR analysis of chemokine mRNA expression in LPLs and MLNs in acute DSS colitis in C57BL/6 and BALB/c mice. Levels of mRNA were normalized to *β*-actin mRNA and presented relative to levels observed in unchallenged BALB/c mice. Data indicate mean ± SD obtained from an experiment involving four animals and representative of two independent experiments. Statistical differences were evaluated by Student's *t*-test. ^∗^*p* < 0.05; ^∗∗^*p* < 0.01. In all assays, the physical (day 0) cell number of immune cell subtypes is 200,000/sample.

To investigate the chemokines involved in mucosal immune response to DSS, RT-PCR was performed on mRNAs isolated from lamina propria and MLN of experimental animals. We observed that DSS challenge of BALB/c mice triggered upregulation of CCR6 (a chemokine for Th17 cells [7]) mRNA, while CCR4, CCR9, and MCP-1/CCL2 mRNA were significantly increased in DSS-challenged C57BL/6 mice (Figure 6). The Th17 chemokine expression pattern in DSS-resistant animals corresponds well with the observed Th17/Treg responses in these animals.

## 4. Discussion

In the present study, we endeavored to obtain insight into the factors that govern differential responses in the intestinal mucosa to noxious stimuli. To this end, we exploited the highly alternative reaction to drinking water DSS in two murine hosts with different genetic backgrounds; *in casu* BALB/c (*H-2^d^*) and C57BL/6 (*H-2^b^*). We establish that although both mouse strains suffer from mucosal damage following the DSS challenge, this damage provokes fulminant inflammation only in C57BL/6 mice. Investigating the nature of the immune response in the two strains, we found that Th1 polarization corresponds to inflammatory damage to the intestine whereas Th17/Treg-mediated immunity relates to tolerance to DSS challenge. We thus propose that differential propensity to develop colitis relates to the intrinsic tendency of immune systems to generate Th1 or Th17/Treg responses, respectively.

The dextran sulfate sodium- (DSS-) induced colitis model is widely used because of its simplicity and the many phenotypical similarities with human IBD [22, 23], particularly UC [24]. Similar to the human situation, where the propensity to develop inflammatory bowel disease is strongly individually dependent, different inbred strains of mice display significantly different susceptibility to DSS colitis [2, 25]. For instance, C3H/HeJ mice are highly susceptible to DSS challenge when compared with other strains of mice, such as C57BL/6. In turn, C57BL/6 (H-2(b)) mice have a higher sensitivity to DSS-induced acute colitis when compared with BALB/c (H-2(d)) mice [21]. In apparent agreement, earlier work of Melgar *c.s.* stressed the effect of genetic background on the outcome of DSS challenge and concluded that C57BL/6 mice offer a more robust experimental colitis model as compared to other strains [26]. Nevertheless, further studies are required to establish the strain-specific elements that govern immune system polarization. In the present study, we aimed to link this dichotomy to the balance between different T helper cell populations. The results indicate that susceptibility to colonic inflammation is linked to a Th1 polarization in the immune system as especially evident from the induction of TNF-*α* and IFN-*γ* production. It is important to note, however, that IFN-*γ* production per se is not a mediator of colitis as experimental colitis can occur in animals deficient in interferon signaling [27], and thus differential IFN-*γ* production is a manifestation of altered immune polarization but unlikely to be causative in this respect. In apparent agreement with a relatively minor role for IFN-*γ* production in experimental colitis is the observation that IFN-*γ* action in general requires other Th1 cytokines, for instance even for canonical antiviral responses [28] IFN-*γ* as such is not sufficient and requires other Th1 cytokines as well, hence, although IFN-*γ* is a valuable marker for immune system polarization, its production is not sufficient for provoking such immune system proliferation. We thus expect that neutralization of IFN-*γ* in our experimental system would only have minor influence. Indeed, it has been reported that neutralization of endogenous IFN-gamma with specific antibodies in DSS-treated BALB/c has only weak ameliorating effects [29]. Nevertheless, we feel that our data indicate that therapies aimed at counteracting Th1 responses might have potential in making colonic tissue resistant to inflammatory responses and ulceration. Conversely, the induction of Th17 and Treg responses may confer protection, also in patients. Indeed, IL-4 confers protection to experimental colitis in DSS-sensitive C57BL/6 mice [30] and further support for this notion can be found in the abject failure of Brodalumab (an IL17-receptor neutralizing antibody) in Crohn's disease and the observation that this antibody increased mucosal inflammation. Also, the success of mucosal interleukin 10 application through its production by genetically modified bacteria supports this notion [21]. Thus, it seems that the observed protective effect of a Th17/Treg-polarized mucosal immune system with respect to colitis observed in the present study might also be relevant for the human situation.

Our data showing an association between Th17 responses and resistance to DSS correspond well to the detrimental effect of Brodalumab in Crohn's disease but may be with other studies that indicate a role for these cells in the pathogenesis of IBD [31]. It is important to note that both mouse strains reacted to DSS with an increase Th17 immunity, only BALB/c mice much more so, and thus this discrepancy may be only apparent. Indeed, IL-17 is a highly pleiotropic cytokine, also produced in the healthy gut and important for intestinal homeostasis. According to our data, most of CD4^+^TCR*αβ*^+^ T cells in the LP are constitutively activated, as indicated by the expression of the activation maker CD69.

Both IL-17A and IL-17F play a key role in the recruitment, activation, and migration of granulocytes and macrophages to induce proinflammatory mediators like IL-6, TNF-α, and different chemokines, such as CCL2 [32, 33]. Despite these apparently mucosal inflammation-stimulating properties, a protective role for Th17 cells is supported by the observation that anti-IL-17A monoclonal antibody treatment aggravates DSS-induced colitis and that blockade of IL-17A in colitis of IL-10 knockout mice does not reduce disease in the absence of IL-6 neutralization [34, 35]. Furthermore, another study demonstrated that adoptive transfer of IL-17A deficient naıve CD4^+^ T cells or transfer of IL-17 receptor deficient T cells to immuno-deficient recipients provokes severe colitis [36]. Collectively, in conjunction with the available literature data, our data argue that although Th17 immunity can have both anti- and proinflammatory effects in the gut, the protective effect appears prevailing.

These protective effects may relate to IL-22, an IL-10 family cytokine, of which Th17 cells are major producers. This cytokine plays an important role in maintaining epithelial homeostasis and promotes intestinal wound healing from acute intestinal injury [37]. IL-22-mediated protective effects were seen in T cell transfer colitis [38]. The current study supports that the protective effects of IL-22 as fulminant in DSS-induced colitis in C57BL/6 mice are associated with a decrease in its mRNA expression. While IL-22 is a potent inflammatory mediator, it may exert a protective influence in both clinical and experimental inflammatory bowel disease. Although understanding the mechanisms that drive uncontrolled inflammation in IBD remains one of the most challenging questions in contemporary experimental medicine, a three-phased view on the pathogenesis of disease is emerging [39]. Following the breakdown of intestinal epithelial barrier function, as the consequence of DSS treatment, a failure to clear the resulting infection early may provoke deleterious immune responses. By stimulating early defense, IL-22 may actually prevent later infection. In apparent support, Pelczar et al. recently published a study in *Science* that production of high levels of IL-22 binding protein and thus neutralizing IL-22 bioactivity aggravates clinical colitis [40]. In this sense, our studies appear to recapitulate clinical IBD fairly well, including a protective role of IL-22 in IBD.

At least in certain patients, IBD involves a failure of immune regulation through defects in Treg cells. Several studies suggest that Treg cell suppression may be more important in the lymph nodes than the site of inflammation [14] and Treg cells express lymph node homing receptors that suppress colitis. Activated Treg cells produce inhibitory cytokines such as IL-10 that may help regulate Th17 cells to acquire a protective rather as a pathogenic phenotype. Hence, we feel that the observed relative upregulation in the BALB/c strain and the associated increase in IL-10 may be an essential element of the protective nature of the immune system in these animals. If this notion is correct, strategies to increase regulatory function should also be considered as a rational therapeutic avenue in human disease.

A factor possibly involved in the recruitment of the apparently protective Th17 cells in BALB/c mice is differential chemokine regulation. The chemokine receptor CCR4 is functionally expressed on human peripheral blood CD4^+^CD25^+^ Treg cells and Th2 cells [41, 42]. The chemokine receptors CCR6 is present on B and T cells as well as dendritic cells. The two CD4^+^ T cell populations with potential protective effects with respect to mucosal inflammation, Treg cells and Th17 cells, both express CCR6. Importantly, in this study, we detected higher CCR6 mRNA expression in DSS-induced colitis BALB/c mice as compared to C57BL/6 mice. Thus, it is tempting to speculate that differential regulation of CCR6 between C57BL/6 and BALB/c mice is responsible for differences in the infiltration of Th17 and Treg to the colon of mice and the associated susceptibility to colitis. Obviously, further work is necessary to substantiate this notion.

In our study, by exploiting the differences between two mouse strains, we were able to show that innate differences in immune polarization steer the response to subsequent noxious stimuli, and it is tempting to speculate that such innate differences in the tendency to develop immune polarization explains why certain patients develop inflammatory bowel disease whereas others in the same environment do not, even if both share IBD risk alleles. The proof of this notion, however, requires identification of the genetic traits that confer the differential tendency BALB/c mice as compared to C57BL/6 mice to develop Th2/Th17/Treg-polarized immunity and Th1-polarized immunity, respectively. If such genetic traits are identified, it should prove highly interesting investigations of the corresponding genetic loci in human genome in relation to IBD development in individuals carrying risk alleles. Such investigations were beyond the scope of the current study but may in the future add substantially to our insight into IBD pathogenesis.

## 5. Conclusion

In conclusion, this study demonstrated that the expression of Th17 and Th17 relative cytokines is higher in BALB/c mice compared with C57BL/6 mice following DSS challenge. BALB/c mice also display concomitant stimulation of Treg cells. These effects correlate with susceptibility to DSS colitis, and thus modulation of Th1, Th2, Th17, and Treg immune responses in acute colitis may be a valuable approach in the treatment and prevention of IBD.

## Supplementary Material

The ARRIVE Guidelines Checklist

## Figures and Tables

**Table 1:** Primer sequences used for real-time PCR.

Gene	Sense	Antisense
IL-2	CCTGAGCAGGATGGAGAATTACA	TCCAGAACATGCCGCAGAG
IL-4	CATCGGCATTTTGAACGAG	TTGGAAGCCCTACAGACGAG
IL-10	C CCCAGAAATCAAGGAGCATT	TCACTCTTCACCTGCTCCAC
IL-12	TGGGA GTACCCTGACTCCTG	GGAACGCACCTTTCTGGTTA
IL-17A	C GCTCCAGAAGGCCCTCAGA	AGCTTTCCCTCCGCATTGA
IL-17F	TCCCACGTGAATTCCAGAAC	ATGGTGCTGTCTTCCTGACC
IL-22	T GACAGGTTCCAGCCCTACAT	CTGGATGTTCTGGTCGTCAC
IL-23	A TGCCCAGCCTGAGTTCTAGT	AGTCAGAGTTGCTGCTCCGT
IFN-*γ*	AAAGACAATCAGGCCATCAG	TGGGTTGTTGACCTCAAACT
TGF-*β*	C TGACGTCACTGGAGTTGTACGG	GGTTCATGTCATGGATGGTGC
TNF-*α*	CCCTCACACTCAGATCATCTTC	GTTGGTTGTCTTTGAGATCCAT
T-bet	CCAGGGAACCGCTTATATGT	CTGGGTCACATTGTTGGAAG
GATA-3	ACAGCTCTGGACTCTTCCCA	GTTCACACACTCCCTGCCTT
STAT-3	TCGTGGAGCTGTTCAGA AAC	GGAAATTTGACCAGCAACCT
ROR-*γ*t	CCACTGCATTCCCAGTTTCT	CGTAGAAGGTCCTCCAGTCG
Foxp3	GGCCCTTCTCCAGGACAGA	GCTGATCATG GCTGGGTTGT
CCR4	TCCTTGGTCTTCTTGTTGGG	GGACAGGACGAACAGCAAAT
CCR5	GTCCTCCTCCTGACCACCTT	GGGTTTAGGCAGCAGTGTGT
CCR6	GTGGTGATGACCTTTGCCTT	GAACGGTAGGGTGAGGACAA
CCR9	TGACTCCACTGCTTCCACAG	GTGCCCACAATGAACACAAG
MCP-1/CCL2	TCCAGAGCTTGAGTGTGACG	TTCAGGGTCAAGGCAAACTT
*β*-actin	TTCCAGCGTTCCTTCTTGGGT	GTTGGCATAGAGGTGTTTACG
